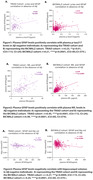# Astrocyte reactivity associates with tau phosphorylation and neurodegeneration before detectable amyloid‐β pathology

**DOI:** 10.1002/alz.093999

**Published:** 2025-01-09

**Authors:** Pampa Saha, Guilherme Povala, Pamela C.L. Ferreira, Livia Amaral, Sarah Abbas, Firoza Z Lussier, Matheus Scarpatto Rodrigues, Markley Oliveira, Nesrine Rahmouni, Cécile Tissot, Carolina Soares, Joseph Therriault, Stijn Servaes, Jenna Stevenson, Chang‐Hyung Hong, Hyun Woong Roh, Helmet T. Karim, Andrea L. Benedet, Nicholas J. Ashton, Henrik Zetterberg, Kaj Blennow, Thomas K Karikari, Sang Joon Kim, Bruna Bellaver, Tharick Ali Pascoal

**Affiliations:** ^1^ University of Pittsburgh, Pittsburgh, PA USA; ^2^ Department of Psychiatry, University of Pittsburgh School of Medicine, Pittsburgh, PA USA; ^3^ Translational Neuroimaging Laboratory, The McGill University Research Centre for Studies in Aging, Montreal, QC Canada; ^4^ Lawrence Berkeley National Laboratory, Berkeley, CA USA; ^5^ Translational Neuroimaging Laboratory, The McGill University Research Centre for Studies in Aging, Montréal, QC Canada; ^6^ Ajou University School of Medicine, Ajou University Hospital, Suwon, Suwon Korea, Republic of (South); ^7^ Ajou University School of Medicine, Suwon, Gyeonggido Korea, Republic of (South); ^8^ Department of Psychiatry and Neurochemistry, Institute of Neuroscience and Physiology, The Sahlgrenska Academy, University of Gothenburg, Mölndal, Gothenburg Sweden; ^9^ University of Gothenburg, Mölndal Sweden; ^10^ Department of Psychiatry and Neurochemistry, Institute of Neuroscience and Physiology, The Sahlgrenska Academy at the University of Gothenburg, Mölndal Sweden; ^11^ Institute of Neuroscience and Physiology, The Sahlgrenska Academy at the University of Gothenburg, Mölndal Sweden; ^12^ Department of Psychiatry, School of Medicine, University of Pittsburgh, Pittsburgh, PA USA; ^13^ Asan Medical Center, University of Ulsan College of Medicine, Seoul Korea, Republic of (South)

## Abstract

**Background:**

Aß plaques are the first detectable signs of AD pathology. Our group recently demonstrated that the astrocyte activation marker, glial fibrillary acidic protein (GFAP), has a pivotal role in the association between Aß burden and tau phosphorylation. However, the role of astrocyte activation in individuals that do not present detectable Aß pathology using biomarkers is still underexplored. Here we sought to understand the association between plasma GFAP with tau phosphorylation and neurodegeneration in Aß negative individuals.

**Method:**

598 study participants [cognitively unimpaired, (CU)=200, and cognitively impaired, (CI)=398] from two cohorts (TRIAD, Canada and BICWALZ, South Korea) were assessed for Aß‐PET, plasma GFAP, p‐tau217, NfL, and hippocampal volume measures. Pearson correlations between plasma GFAP and plasma p‐tau217, plasma NfL along with hippocampal volume was performed in Aß‐PET negative individuals.

**Result:**

Remarkably, we found that plasma GFAP levels positively correlate with plasma p‐tau217 levels in Aß‐PET negative individuals in both cohorts [TRIAD cohort: r=0.23, p=0.01; BICWALZ cohort: r=0.21, p=0.0001; Figure: 1A, B]. Further, the correlation between plasma GFAP with plasma NfL has also been found to be positively correlated among the two cohorts [TRIAD cohort: r=0.45, p=0.0001; BICWALZ cohort: r=0.49, p=0.0001; Figure: 2A, B]. We also observed that hippocampal volume, as a neurodegeneration marker, reveals a significant negative Pearson correlation with plasma GFAP levels in Aß‐PET negative human individuals from both the cohorts [TRIAD cohort: r=‐0.27, p=0.0009; BICWALZ cohort: r=‐0.24, p=0.0001; Figure: 3A, B].

**Conclusion:**

Our study provides evidence from research and clinical cohorts that astrocyte reactivity may be associated with very early tau phosphorylation and neurodegeneration in the absence of Aß pathology.